# Mechanisms of Copper Tolerance, Accumulation, and Detoxification in the Marine Macroalga *Ulva compressa* (Chlorophyta): 20 Years of Research

**DOI:** 10.3390/plants9060681

**Published:** 2020-05-27

**Authors:** Alejandra Moenne, Melissa Gómez, Daniel Laporte, Daniela Espinoza, Claudio A. Sáez, Alberto González

**Affiliations:** 1 Laboratory of Marine Biotechnology, Faculty of Chemistry and Biology, University of Santiago of Chile, Santiago 916000, Chile; melissa.gomez@usach.cl (M.G.); daniel.laporte@usach.cl (D.L.); daniela.espinoza@usach.cl (D.E.); 2 Laboratory of Aquatic Environmental Research, Centro de Estudios Avanzados, Universidad de Playa Ancha, Viña del Mar 2520000, Chile; claudio.saez@upla.cl; 3 Hub Ambiental UPLA, Universidad de Playa Ancha, Valparaíso 2390302, Chile

**Keywords:** ascorbate, copper, glutathione, marine alga, metallothioneins, phytochelatins, Ulva compressa

## Abstract

Copper induces an oxidative stress condition in the marine alga *Ulva compressa* that is due to the production of superoxide anions and hydrogen peroxide, mainly in organelles. The increase in hydrogen peroxide is accompanied by increases in intracellular calcium and nitric oxide, and there is a crosstalk among these signals. The increase in intracellular calcium activates signaling pathways involving Calmodulin-dependent Protein Kinases (CaMKs) and Calcium-Dependent Protein Kinases (CDPKs), leading to activation of gene expression of antioxidant enzymes and enzymes involved in ascorbate (ASC) and glutathione (GSH) synthesis. It was recently shown that copper also activates Mitogen-Activated Protein Kinases (MAPKs) that participate in the increase in the expression of antioxidant enzymes. The increase in gene expression leads to enhanced activities of antioxidant enzymes and to enhanced levels of ASC and GSH. In addition, copper induces an increase in photosynthesis leading to an increase in the leve of Nicotinamide Adenine Dinucleotide Phosphate (NADPH). Copper also induces an increase in activities of enzymes involved in C, N, and S assimilation, allowing the replacement of proteins damaged by oxidative stress. The accumulation of copper in acute exposure involved increases in GSH, phytochelatins (PCs), and metallothioneins (MTs) whereas the accumulation of copper in chronic exposure involved only MTs. Acute and chronic copper exposure induced the accumulation of copper-containing particles in chloroplasts. On the other hand, copper is extruded from the alga with an equimolar amount of GSH. Thus, the increases in activities of antioxidant enzymes, in ASC, GSH, and NADPH levels, and in C, N, and S assimilation, the accumulation of copper-containing particles in chloroplasts, and the extrusion of copper ions from the alga constitute essential mechanisms that participate in the buffering of copper-induced oxidative stress in *U. compressa.*

## 1. Introduction

### 1.1. Mechanisms of Copper Tolerance in Plants and Marine Macroalgae

Copper is an essential heavy metal, as well as iron and zinc and others, since it is required for the functioning of several proteins and enzymes such as plastocyanin, cytochrome c oxidase, laccase, Cu/Zn superoxide dismutase, and several other oxidases and dehydrogenases [1,2]. However, these essential heavy metals in excess produce an oxidative stress condition that oxidizes biological macromolecules such as proteins, fatty acids, and nucleic acids [1,2]. On the other hand, there are non-essential heavy metals such as cadmium, lead, mercury, silver, and arsenic that are not required for protein or enzyme function and these metals are toxic even in very low amounts. Interestingly, cadmium is an essential heavy metal in marine phytoplankton since it is required for carbonic anhydrase activity [3], but in animals and plants, cadmium is a non-essential heavy metal.

Heavy metals, and copper in particular, produce oxidative stress due to the increase in the synthesis of reactive oxygen species (ROS), such as superoxide anions, hydrogen peroxide, and hydroxyl radicals, in organelles and in the cytoplasm [4]. The oxidative stress condition is initially buffered by the activation of antioxidant enzymes and in the synthesis of antioxidant molecules that are the substrates of antioxidant enzymes [4]. Antioxidant enzymes are superoxide dismutase (SOD), which dismutates superoxide anions into hydrogen peroxide, as well as catalase (CAT), glutathione peroxidase (GP), ascorbate peroxidase (AP), and peroxiredoxin (PRX) that convert hydrogen peroxide into water and oxygen. In photosynthetic organisms, AP is coupled to dehydroascorbate reductase (DHAR) and glutathione reductase (GR) constituting the Halliwell–Asada–Foyer cycle. AP uses ascorbate (ASC) as substrate and produces dehydroascorbate (DHA), DHA is reduced to ASC by DHAR that uses glutathione (GSH) as substrate and produces oxidized glutathione (GSSG), and GSSG is reduced to GSH by GR that uses nicotinamide adenine dinucleotide phosphate (NADPH) as final reducing power [4].

The antioxidant molecules ASC and GSH can also directly reduce ROS. ASC is synthesized from glucose 6-P and involves several enzymes of which the latest are L-galactose dehydrogenase (L-GDH) and L-galactono 1,4 lactone dehydrogenase (L-GLDH) [5]. ASC is also the substrate of de-epoxidases in the xantophyll cycle that protects thylakoid membranes from lipid oxidation [6,7]. ASC can also prevent photo-inhibition by donating electrons to the electron transport chain in plants exposed to high light [6,7]. On the other hand, GSH is constituted by the amino acids glutamate, cysteine and glycine, and it is synthesized by the enzymes γ-glutamyl-cysteinyl synthase (γ-GCS) and glutathione synthase (GS), and both enzymes require ATP [8]. GSH is also required for glutathionylation of xenobiotics or endobiotics in phase II reactions, rendering the hydrophobic molecules such as polycyclic aromatic hydrocarbons, linear hydrocarbons, lipid hormones, cholesterol, and other lipid compounds more soluble in water [9]. 

In plants, it has been shown that the aquatic plant *Ceratophyllum demersum* cultivated with 2 and 4 μM of copper for 24 h displayed a decrease in chlorophyll content, an increase in lipoperoxides and ion leakage, an increase in SOD, AP, and CAT activities, and the synthesis of GSH [10]. *Arabidopsis thaliana* cultivated with 0 to 300 μM of copper for 7 days showed a decrease in leaf biomass, an accumulation of copper in leaves, mainly with 150 and 300 μM, an increase in the level of superoxide anions, hydrogen peroxide, and hydroxyl radicals, an increase in activities of SOD and guaiacol peroxidase (POX), but a decrease in CAT activity [11]. In addition, *A. thaliana* cultivated with 0 to 100 μM of copper for 0, 3, and 7 d showed the synthesis of ASC and GSH mainly with higher concentrations of copper [12]. *Zea mays* plants cultivated with 0 to 100 μM of copper for 24 h showed an increase in hydrogen peroxide in leaves and an increase in SOD, CAT, and AP activities as well as in the activity of the MAPK ZmMPK3 [13]. The inhibition of ZmMPK3 with PD98059 significantly blocked its activity leading to a decrease in SOD, CAT, and AP activities, indicating that ZmMPK3 is involved in the activation of the antioxidant system [13]. Thus, plants exposed to copper excess showed an oxidative stress condition, the activation of the antioxidant system, and the synthesis of antioxidant compounds such as ASC and GSH. 

In marine macroalgae, it has been shown that the red macroalga *Gracilaria tenuistipitata* cultivated with 0 to 5 μg L^−1^ of copper for 4 d displayed a decrease in growth and in protein content, an increase in the level of carbonylated proteins and lipoperoxides, and an increase in activities of SOD, CAT, and AP as well as in the level of the antioxidant molecules luthein and β-carotene [14]. The brown macroalga *Ectocarpus siliculosus* cultivated with 2.4 μM of copper for 10 d showed an increase in hydrogen peroxide and lipoperoxides, an increase in SOD, CAT, and AP activities, and an increase in ASC and GSH levels [15]. In addition, *E. siliculosus* displayed a decrease in growth and an increase in intracellular copper and in the level of transcripts encoding enzymes involved in GSH synthesis, γ-GCS and GS [16]. Moreover, *E. siliculosus* cultivated with 1.8 and 2.4 μM of copper for 0 to 8 h showed an increase in photosynthesis with 0.8 μM, but a decrease in photosynthesis with 2.4 μM of copper [17]. In addition, a microarray analysis was performed with total RNA of *E. siliculosus* cultivated with 2.4 μM of copper for 4 and 8 h and showed inhibition of inositol-dependent signaling pathway, the activation of oxylipin-dependent signaling pathway, and the modulation in expression of several transcription factors [17]. Furthermore, copper induced an increase in the level of transcripts encoding antioxidant enzymes, including a bromo-dependent peroxidase and several ABC transporters, and a decrease in the level of transcripts encoding enzymes involved in N assimilation. Moreover, a metabolomic analysis revealed an increase in the level of free fatty acids in *E. siliculosus* treated with copper [17]. Thus, marine macroalgae exposed to copper excess showed an oxidative stress condition, the activation of antioxidant enzymes, and the synthesis of antioxidant molecules such as ASC and GSH. 

### 1.2. Mechanisms of Copper Accumulation in Plants and Marine Macroalgae

Another mechanism to cope with heavy metal-induced oxidative stress is the synthesis of cysteine-rich peptides and proteins that sequester heavy metals, such as phytochelatins (PCs) and metallothioneins (MTs) [18]. PCs are formed by condensation of GSH units (n = 2–12) and they are synthesized by the enzyme phytochelatin synthase (PCS). PCs are synthesized in yeast, algae, nematodes, and plants, but not in animals, and plant genomes encode one or two genes of PCS [19]. PCs can sequester mono- or divalent cations such as copper, zinc, cadmium, arsenite, and arsenate [20]. On the other hand, metallothioneins are gene-encoded small proteins (around 10 kDa) containing a high percentage of cysteines (20%–30%) as well as glycine and alanine [18]. MTs can sequester divalent and monovalent cations such as copper, zinc, cadmium, lead, mercury, silver, arsenite, and arsenate [18]. MTs are present in cyanobacteria, protists, fungi, nematodes, algae, plants, and animals [21,22,23]. In animals, there are four MTs; in fish, two MTs; in invertebrates, mainly two MTs; and in yeast, two MTs, namely CUP-1 and CRS-5 [21]. In plants, there are mainly six MTs as it has been shown in *Arabidopsis thaliana* and *Populus trichocarpa x deltoides* [23]. In marine brown and red macroalgae, there is only one MT, and until recently the only cloned and expressed MT was the brown macroalga *Fucus vesiculosus* MT [23,24]. Recently, three MTs were identified in the green macroalga *Ulva compressa* [25]. 

In plants, it has been shown that the aquatic plant *Hydrilla verticillata* cultivated with 0 to 25 μM of copper for 0 to 7 d showed an increase in non-protein thiols (GSH + PCs) with concentrations of 0.1 to 5 μM, but a decrease in non-protein thiols with 25 μM of copper [26]. The copper-tolerant plant *Silene cucubalus* cultivated with 0.01 to 1000 μM of copper did not show an increase in PCs with concentrations lower than 100 μM [27]. Alfalfa (*Medicago sativa*) plants did not show the synthesis of PCs in response to 0 to 100 μM of copper after 6 days [28]. Thus, copper excess does not necessarily induce PC synthesis in plants. Regarding MTs, the *A. thaliana* MTs, namely MT1a, MT2a, MT2b, MT3, and MT4a, were expressed in the yeast *Saccharomyces cerevisiae* that lack CUP-1 MT, and they allowed copper accumulation [29]. The lack of MT1a, but not MT2b, produced a 30% lower accumulation of copper in *Arabidopsis* leaves, and the expression of MT1a in the double mutant that lacks MT1a/MT1b restored copper accumulation [29]. The quadruple mutant of *A. thaliana* that lacks MT1a/MT2a/MT2b/MT3 accumulated 45% less copper than the control [30]. Furthermore, the overexpression of MT1 of the copper-accumulator plant *Elsholtzia haichowensis* in tobacco plants allowed copper accumulation in roots [31]. In this regard, the six MTs of *A. thaliana* and the four MTs of *Noccacea caerulescens* expressed and anchored to the inner face of the plasma membrane through a myristoil tail fused to GFP and fused to the C-terminal region of MTs allowed the accumulation of copper and other heavy metals in yeast [32]. Thus, copper excess normally induced the synthesis of MTs in plants allowing copper accumulation. 

In marine macroalgae, a copper-tolerant strain of *E. siliculosus*, Es524, cultivated with 0 to 2.4 μM of copper for 10 d showed an accumulation of copper and an increase in the PC2 level, but a decrease in the PC3 level. A more copper-sensitive strain of *E. siliculosus*, the LIA strain, did not show an increase in the synthesis in PC2 or PC3 levels [16]. In addition, the brown macroalga *Fucus serratus* collected in a heavy metal-contaminated site showed an increase in PC2, PC3, and PC4 levels [33]. On the other hand, the amount of transcripts encoding the MT of the brown alga *Fucus vesiculosus* increased in response to copper excess [24]. The overexpression of *Fucus* MT in *Escherichia coli* allowed the accumulation of arsenite and arsenate, but not cadmium, zinc, or lead [34]. It was recently shown that three MTs of the green alga *U. compressa* allowed the accumulation of copper and zinc in *E. coli* [25]. Thus, the excess of copper and other heavy metals induced the synthesis of PCs and MTs in marine macroalgae allowing heavy metal accumulation.

## 2. Mechanisms of Copper Tolerance in the Marine Macroalga *U. compressa*

The marine alga *U. compressa* is the dominant species in coastal sites of northern Chile contaminated with copper and other heavy metals [35,36]. Regarding the mechanisms of copper tolerance in *U. compressa*, it was initially shown that the alga cultivated with a sub-lethal concentration of copper (10 μM) for 7 d showed an increase in hydrogen peroxide level at 2, 3, and 12 h and a sustained increase in superoxide anion beginning on day 3 until day 7 [37,38]. In addition, the production of hydrogen peroxide and superoxide anion occur mainly in mitochondria and chloroplasts [37]. The alga cultivated with 10 μM for 7 d showed an increase in activities of antioxidant enzymes, mainly SOD, AP, GR, and the detoxification enzyme glutathione-S-transferase (GST) [37,38]. In addition, the alga cultivated with 10 μM of copper for 7 d showed the synthesis of ASC, GSH, and PCs [39]. Transcripts encoding antioxidant enzymes such as AP, DHAR, and GR as well as enzymes involved in ASC and GSH synthesis were increased, indicating that the higher enzyme activities are due to an increase in gene expression [39,40]. 

**Figure 1:**
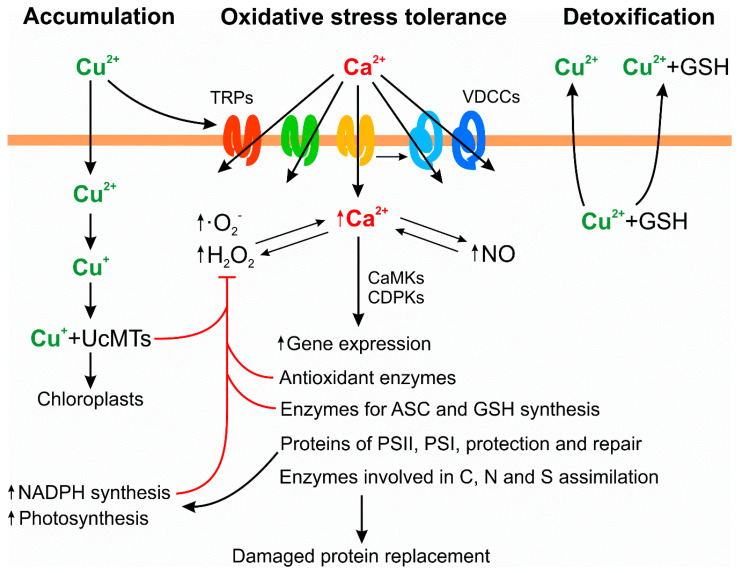
Scheme of copper-induced extracellular calcium entry through transient receptor potential (TRP) channels and voltage-dependent calcium channels (VDCCs) leading to the increases in intracellular calcium which crosstalk with increases in hydrogen peroxide and nitric oxide (NO). The increases in intracellular calcium activate CaMKs and CDPKs that, in turn, activate gene expression leading to enhance activities of antioxidant enzymes, ASC and GSH levels, proteins of photosystems (PSII and PSI) and proteins involved in protection and repair of PS, and enzymes involved in C, N, and S assimilation. The increase in photosynthesis enhances NADPH levels that coupled to the increase in ASC and GSH levels and in activities of antioxidant enzymes participate in the buffering of copper-induced oxidative stress. In addition, the binding of copper ions to UcMTs, the accumulation of copper-containing particles in chloroplasts, and the copper extrusion with GSH also participate in the buffering of oxidative stress. TRPs are indicated in red, green, and yellow; VDCCs are indicated in light and dark blue; red arrows indicate inhibitory processes; and black arrows indicate activation processes.

In addition, increases in intracellular calcium were observed at 2, 3, and 12 h of copper exposure [41] and calcium was released from endoplasmic reticulum (ER) through the ryanodine receptor, IP_3_-dependent channels, and Nicotinic Acid Adenine nucleotide Diphosphate (NAADP)-dependent channels [38]. It was further determined that an increase in intracellular calcium induced the production of hydrogen peroxide in organelles, and vice versa, and the increase in intracellular calcium increased NO production, and vice versa, but the increase in hydrogen peroxide only increased NO production [38]. It was shown that the increase in intracellular calcium activates Krebs cycle enzymes that produce NADH that, in turn, activates electron transport chain in mitochondria leading to an increase in hydrogen peroxide production [38]. The increase in intracellular calcium leads activation of CaMs and, thus, CaMKs, as well as in CDPK signaling pathways leading to an increase in gene expression of antioxidant enzymes such as SOD, AP, and PRX [38]. Thus, there is a crosstalk among intracellular calcium, hydrogen peroxide, and NO, and the increase in intracellular calcium activates CaMKs and CDPKs leading to activation of gene expression of antioxidant enzymes (see the scheme in Figure 1). 

It was further shown that the intracellular calcium release at 2, 3, and 12 h of copper exposure required the entry of extracellular calcium through voltage-dependent calcium channels (VDCCs), indicating that there is a calcium-induced calcium release response in the alga exposed to copper excess [42]. Extracellular calcium entry though VDCCs required the previous activation of transient receptor potential (TRP) channels such as TRPA1, TRPC5, and TRPV1 at 5, 8, and 11 to 13 min of copper exposure. In addition, there was a concomitant entry of extracellular copper at 5, 8, and 11 to 13 min of copper exposure that induced membrane depolarization at 5, 8, and 11 to 13 min of copper exposure [43]. Additional membrane depolarization were detected at 80 and 86 min and at 5 and 9 h; the depolarization at 80 and 86 min were due to copper ion entry, whereas those at 5 and 9 h and the divalent ion that entry at 5 and 9 h was not identified [44]. VDCC activation at 2 h was dependent on TRP activation at 4, 8, 11–13, 80, and 86 min, VDCC activation at 3 h was dependent on TRP activation at 80 and 86 min, and VDCC activation at 12 h was dependent on TRP activation at 5 and 9 h [44]. Thus, copper-induced activation of TRPs leads to the activation of VDCCs allowing extracellular calcium entry and intracellular calcium release.

On the other hand, it was determined that copper induced the release of phenylalanine, tryptophan, methionine, and arginine with maximal levels at 2, 2.5, 3.5, and 4.5 min, respectively, after copper addition, serotonin with a maximal level at 3 min, and adrenalin with maximal levels at 1.5, 3, and 5 min [45]. The release of amino acids and neurotransmitters activates glutamate-, serotonin- and adrenalin-like receptors leading to TRP activation at 5, 8, and 11 to 13 min [45]. The release of amino acids and neurotransmitters was dependent on the initial activation of TRP channels at time 0, after 1 min and after 2 min of copper addition [45]. Thus, activation of TRPs at time 0, 1, and 2 min is required for the release of amino acids, serotonin, and adrenalin that activate glutamate-, serotonin- and adrenalin-like receptors that, in turn, activate TRPs at 5, 8, and 11 to 13 min leading to activation of VDCCs and the entry of extracellular calcium and intracellular calcium release.

It was additionally shown that the increase in gene expression involves the mitogen-activated protein kinases (MAPKs) signaling pathway [40]. The MAPKs, JNK, ERK, and p-38 are involved in the regulation of gene expression of antioxidant enzymes such as SOD, CAT, AP, DHAR, and GR [46], photosynthetic parameters, and copper accumulation, and these MAPKs showed a crosstalk among them [47]. Thus, activation of gene expression involves CaMK, CDPK, and MAPK signaling pathways. 

## 3. Mechanisms of Copper Accumulation in the Marine Macroalga *U. compressa*

It was initially shown that *U. compressa*, collected in control sites of northern and central Chile and in copper-polluted sites of northern Chile, displayed copper accumulation in its tissue, an increase in AP activity, and the synthesis of ASC that was accumulated as DHA [35]. In addition, the level of DHA linearly correlated with the concentration of copper in seawater. Then, the alga was cultivated with increasing concentrations of copper, such as 2.5, 5, 7.5, and 10 μM, for 0 to 12 d. The level of intracellular copper linearly correlated with copper concentration in seawater, and with the elapsed time of culture [48]. The level of GSH increased with the lower concentrations of copper (2.5 and 5 μM), from day 1 until day 12, and the level of PC2 and PC4 increased with the higher concentrations of copper (7.5 and 10 μM) from day 1 until day 12 [48]. Interestingly, the level of total non-protein thiols (GSH + PCs) correlated with intracellular copper levels observed at day 12. Moreover, the level of transcripts encoding the MTs, namely UcMT1, UcMT2, and UcMT3, increased from day 3 until day 12 [48]. Thus, in acute condition of copper excess the alga initially synthesized GSH and PCs and further showed an increase in UcMT expression, suggesting that GSH, PCs, and UcMTs participate in copper accumulation in *U. compressa*. 

It was initially shown using transcriptomic analyses that *U. compressa* exposed to 10 μM of copper for 0 and 24 h encoded seven potential UcMTs and that the expression of these MTs was upregulated [40]. Then, transcripts encoding UcMT1, UcMT2, and UcMT3 were cloned and sequenced and showed that UcMT1 is a 8.2 kDa protein containing 29.4% of cysteines arranged as 3 CC and 7 CXC motifs; UcMT2 is a 9.05 kDa protein containing 30% of cysteines arranged as 3 CC, 7 CXC, and 1 CXXC motifs; and UcMT3 is a13.4 kDa protein containing 24% of cysteines arranged as 7 CC and 7 CXC motifs [25]. Interestingly, UcMTs showed homology with marine invertebrate MTs and with the terrestrial invertebrate *Caenorhabidtis elegans* MTs. Surprisingly, the UcMTs’ amino acid sequences were not closely related with MTs from red or brown marine macroalgae. The open reading frames (ORFs) of UcMTs were cloned in an expression vector that allow the synthesis of a fusion protein containing in N-terminal position a GST of the flatworm *Shistosoma japonicum* followed by UcMT [25]. Importantly, *S. japonicum*’s GST contains a single cysteine in its amino acid sequence and, thus, is unable to bind heavy metal ions. GST-UcMTs were overexpressed in *E. coli* and their overexpression allowed the accumulation of proteins having the predicted sizes and GST tail that was detected using a specific antibody [25]. More importantly, UcMTs overexpressed in *E. coli* allowed the accumulation of copper and zinc in vivo; UcMT1 and UcMT2 allowed a higher accumulation of copper compared with UcMT3 and control bacteria, and the three UcMTs allowed similar accumulation of zinc levels [25]. Thus, UcMTs are real MTs that bind copper and zinc in vivo and they are probably involved in copper accumulation in *U. compressa*. 

*U. compressa* was collected in control sites of northern and central Chile and in copper-polluted sites of northern Chile and the levels of copper in seawater, intracellular copper, GSH, PCs, and MT transcripts and proteins were analyzed [49]. The level of copper in seawater was different than that in copper-polluted sites, whereas the level of intracellular copper was similar, suggesting that an extrusion mechanism is operating in algae of copper-polluted sites. Interestingly, the level of GSH was undetectable and the level of PCs was very low compared to that of PCs detected in algae cultivated with increasing concentrations of copper for 0 to 12 d [48]. In contrast, the levels of transcripts encoding UcMT1 and UcMT2 were increased compared to those of control sites, whereas the UcMT3 level was not increased [49]. In addition, protein extracts obtained from the alga of a control site and a copper-polluted site were heated at 70 °C for 10 min and the non-denatured proteins present in the supernatant were analyzed in a denaturant polyacrylamide gel. Interestingly, a single protein of apparent MW of 11 kDa was observed that may correspond to UcMT1 and/or UcMT2 (Espinoza et al., in preparation). In the future, UcMT will be purified and will be identified using antibodies prepared against UcMT1 and UcMT2. On the other hand, the alga collected in a control site and in a copper-polluted site was visualized by transmission electron microscopy (TEM, Titan, TemoFisher Scientific, USA) and electron-dense particles were analyzed to detect copper using energy-dispersive X-ray spectrometry (EDXS, Brucker, Germany). In addition, the alga was cultivated with 10 μM of copper for 5 d, and under control conditions without copper addition. Cells of algae from copper-polluted sites and cells of algae cultivated with copper showed electron-dense particles containing copper, whereas those in algae of control sites or algae cultivated under control conditions did not contain copper [49]. Interestingly, electron-dense particles containing copper were located only in chloroplasts. It is important to mention that copper-containing particles showed an amorphous form, and not a crystalline form, suggesting that copper ions may be bound to a protein. Thus, it is possible that copper accumulation in *U. compressa* is due to the binding of copper ions to UcMT1 and/or UcMT2, and these copper-containing particles are located in chloroplasts. 

## 4. Mechanisms of Copper Detoxification in the Marine Macroalga *U. compressa*

An additional mechanism of copper tolerance was detected in the alga cultivated in synthetic seawater with 10 μM of copper for 5 d and then introduced into synthetic seawater without copper for 3 d. It was observed that the level of intracellular copper decreased, whereas the level of copper in seawater increased, indicating that a copper extrusion mechanism is operating in the alga exposed to copper excess [48]. In addition, equimolar amounts of GSH regarding copper levels were detected in the culture medium, and no peptides or small proteins were observed [48]. This indicates that copper and GSH are extruded from the alga and suggests that GSH may be required for copper extrusion and/or that GSH can chelate copper inhibiting the re-entry into the alga (see the scheme in Figure 1). In this sense, it has been shown that microalgae and macroalgae exudate cysteine and/or GSH in response to copper excess and that exudation of cysteine and/or GSH may inhibit the re-entry of copper inside the cells [50,51]. 

## 5. Additional Mechanisms of Copper Tolerance in the Marine Macroalga *U. compressa*

Transcriptomic analyses were performed with alga cultivated with 10 μM of copper for short times such as 0, 3, 6, 12 and 24 h and longer times such as 0, 1, 3 and 5 d in order to find new UcMTs and probably a PCS [52,53]. Transcriptomic analyses performed for long times allowed the identification of two potential new UcMTs, but not a PCS. Transcriptomic analyses performed at shorter times showed that transcripts, that are differentially expressed, encode several subunits of photosystem (PS) II and PSI and proteins and enzymes involved in repair of PSII and protection of PSI, and the level of these transcripts was increased [52]. In addition, net photosynthesis was enhanced in the alga cultivated from 3 h to 24 h with copper compared to controls. Transcriptomic analyses performed at longer times also showed the increase in transcripts encoding subunits of PSII and PSII and proteins and enzymes involved in repair and protection of PSII and PSI. In addition, transcripts differentially expressed showed an increase in the level of transcripts encoding several subunits of mitochondrial electron transport chain as well as those encoding enzymes involved in C, N, and S assimilation [53]. Moreover, net photosynthesis and respiration were increased in the alga cultivated with 10 μM of copper for 0 to 5 d as well as the activities of enzymes involved in C, N, and S assimilation [53]. The concomitant increases in photosynthesis, respiration, and C, N, and S assimilation represent an exceptional mechanism of copper tolerance since the mechanism has not been described in plants or algae exposed to copper excess. Thus, an additional mechanism of copper tolerance was identified in *U. compressa* corresponding to the increase in photosynthesis that may produce an enhanced level of NADPH. In fact, it was recently determined that *U. compressa* cultivated with copper showed an increase in NADPH levels, which is mainly due to the increase in photosynthesis [54]. The increased levels of NADPH may favor the activity of the antioxidant enzyme such as GR and the increase in ASC and GSH levels may favor the activities of antioxidant enzymes such as AP and DHAR. In addition, the increase in C, N, and S assimilation may participate in the replacement of proteins damaged by copper-induced oxidative stress. Thus, the increase in antioxidant enzymes activities, ASC, GSH, and NADPH levels, and enhanced C, N, and S assimilation are processes that contribute to copper homeostasis and attenuation of oxidative stress (see the scheme in Figure 1). Finally, it is important to mention that sequencing of the *U. compressa* genome is in progress and the annotation of genes will allow the identification of new UcMTs and at least one PCS in the future. 

## 6. Conclusions

Copper induces an oxidative stress condition due to the production of superoxide anions and hydrogen peroxide, mainly in organelles, in *U. compressa.* The increase in hydrogen peroxide occurred concomitantly with increases in intracellular calcium and nitric oxide and there was a crosstalk among these signals. The increase in intracellular calcium activates CaMKs and CDPKs, inducing the activation of gene expression of antioxidant enzymes and enzymes involved in ASC and GSH synthesis. It was recently shown that copper also activates Mitogen-Activated Protein Kinases (MAPKs) that participate in the increase in the expression of antioxidant enzymes. The increase in gene expression leads to enhanced activities of antioxidant enzymes and in ASC and GSH levels. In addition, copper induces an increase in net photosynthesis, leading to an enhanced level of NADPH. Moreover, copper induces an increase in activities of enzymes involved in C, N, and S assimilation, allowing the replacement of proteins damaged by oxidative stress. The accumulation of copper in acute exposure involves an increase in GSH, PC, and UcMT levels, whereas the accumulation of copper in chronic exposure involves only UcMTs. Copper induces the accumulation of copper-containing particles that may correspond to UcMTs bound to copper ions, in chloroplasts. On the other hand, it was shown that copper is extruded from the alga concomitantly with an equimolar amount of GSH. Thus, the increase in activities of antioxidant enzymes, the enhanced production of ASC, GSH, and NADPH, the increased C, N, and S assimilation, the accumulation of copper-containing particles in chloroplasts, and the extrusion of copper from the alga are essential mechanisms that participate in the buffering of copper-induced oxidative stress in *U. compressa*. 

## Abbreviations

ROS:	reactive oxygen species
AP:	ascorbate peroxidase
DHAR:	dehydroascotbate reductase
GR:	glutathione reductase
CAT:	catalase
GP:	glutathione peroxidase
PRX:	peroxiredoxin
ASC:	ascorbate
GSH:	reduced glutathione
GSSG:	oxidized glutathione
NADPH:	nicotinamide adenine dinucleotide phosphate
PC:	phytochelatin
MT:	metallothionein
TRP:	transient receptor potencial
VDCC:	voltage-dependent calcium channel
CaM:	calmodulin
CaMK:	calmodulin-dependent protein kinase
CDPK:	calcium-dependent protein kinase
MAPK:	mitogen-activated protein kinase
